# Waning cellular immune responses and predictive factors in maintaining cellular immunity against SARS-CoV-2 six months after BNT162b2 mRNA vaccination

**DOI:** 10.1038/s41598-023-36397-6

**Published:** 2023-06-13

**Authors:** Takashi Ishii, Kensuke Hamada, Daisuke Jubishi, Hideki Hashimoto, Koh Okamoto, Naoko Hisasue, Mitsuhiro Sunohara, Minako Saito, Takayuki Shinohara, Marie Yamashita, Yuji Wakimoto, Amato Otani, Mahoko Ikeda, Sohei Harada, Shu Okugawa, Kyoji Moriya, Shintaro Yanagimoto

**Affiliations:** 1 Division for Health Service Promotion, The University of Tokyo, Hongo 7-3-1, Bunkyo, TokyoTokyo, 113-8655 Japan; 2 Department of Infectious Diseases, The University of Tokyo Hospital, Tokyo, Japan; 3 Department of Infection Control and Prevention, The University of Tokyo Hospital, Tokyo, Japan

**Keywords:** Immunology, Medical research

## Abstract

Several clinical trials have shown that the humoral response produced by anti-spike antibodies elicited by coronavirus disease 2019 (COVID-19) vaccines gradually declines. The kinetics, durability and influence of epidemiological and clinical factors on cellular immunity have not been fully elucidated. We analyzed cellular immune responses elicited by BNT162b2 mRNA vaccines in 321 health care workers using whole blood interferon-gamma (IFN-γ) release assays. IFN-γ, induced by CD4 + and CD8 + T cells stimulated with severe acute respiratory syndrome coronavirus 2 (SARS-CoV-2) spike epitopes (Ag2), levels were highest at 3 weeks after the second vaccination (6 W) and decreased by 37.4% at 3 months (4 M) and 60.0% at 6 months (7 M), the decline of which seemed slower than that of anti-spike antibody levels. Multiple regression analysis revealed that the levels of IFN-γ induced by Ag2 at 7 M were significantly correlated with age, dyslipidemia, focal adverse reactions to full vaccination, lymphocyte and monocyte counts in whole blood, Ag2 levels before the second vaccination, and Ag2 levels at 6 W. We clarified the dynamics and predictive factors for the long-lasting effects of cellular immune responses. The results emphasize the need for a booster vaccine from the perspective of SARS-CoV-2 vaccine-elicited cellular immunity.

## Introduction

Coronavirus disease 2019 (COVID-19), which is caused by severe acute respiratory syndrome coronavirus 2 (SARS-CoV-2), was the cause of the global pandemic in 2019. The WHO reported that over six million people have died worldwide, mainly due to severe viral pneumonia^1^. Highly effective mRNA vaccines against SARS-CoV-2 were rapidly developed and have contributed to curbing the spread of infection and reducing the severity of disease^2,3^. Immune responses following SARS-CoV-2 infection or vaccination have been studied worldwide, including both humoral and cellular responses^4–6^. The humoral response is characterized by B-cell-associated immunity after vaccination, with the production of antibodies against the spike glycoprotein S1 subunit, which is responsible for the binding of the virus to the angiotensin-converting enzyme 2 receptor of host cells and entry of the virus into the host cells. Antibody neutralizing SARS-CoV-2 is highly predictive of immune protection^7^ and correlates with anti-S1 antibody levels, which can be measured using an established platform; therefore, anti-S1 antibody levels are a good biomarker of host defense through humoral immunity against SARS-CoV-2 infection^8^.

The T-cell-mediated response, defined as a cellular immune response, is also essential for host defense, removing infected cells and limiting infection. The SARS-CoV-2-specific T-cell response can be measured by an interferon-gamma (IFN-γ) release assays (IGRA) using SARS-CoV-2 antigens that stimulate CD4^+^ T cells and/or CD8^+^ T cells^9,10^. Cellular immunity, as well as humoral immunity^11,12^, is indispensable for controlling SARS-CoV-2 infection and has been shown to be involved in past COVID-19 infection or severity^13,14^. Vaccination induces rapid antigen-specific CD4 + T-cell responses in naive subjects after the first vaccination and is associated with coordinated humoral and cellular immunity^6^. In our previous prospective observational cohort study, two doses of BNT162b2 mRNA vaccines resulted in an increase in IFN-γ titers measured by IGRA, accompanied by high anti-S1 antibody levels^15^. Notably, spike-specific CD4 + and CD8 + T-cell responses with extensive cross-reactivity against both the Delta and Omicron variants have been detected in BNT162b2 mRNA vaccines, despite the substantially reduced levels of SARS-CoV-2 neutralizing antibodies^16^.

The question remains as to whether and how both immune responses vary after infection or full vaccination, especially over time. In SARS-CoV-2 infection, although the rapid decline of the humoral immune response over time has been documented by several studies, it is unclear whether the cellular immune response declines within a few months or remains relatively stable as a memory response^17,18^. Several studies have demonstrated that neutralizing or anti-spike antibody levels decline rapidly and may be involved in susceptibility to SARS-CoV-2 infection^19,20^. Some studies have demonstrated that cellular immune responses also wane 6 months after vaccination^21,22^. However, an analysis of predictive factors for maintaining strong cellular immune responses and the dynamics and durability of cellular immune responses in a large population is still needed.

We investigated the dynamics of anti-spike antibody titers and IFN-γ levels measured via IGRA in health care workers after BNT162b2 mRNA vaccination as a follow-up to our previous prospective observational study^15^. Focusing on IGRA, we clarified the dynamics of IFN-γ levels until 6 months after vaccination and the characteristics of people with relatively high titers of IFN-γ who showed persistent cellular immune responses after vaccination.

## Results

### Characteristics of the study participants

A total of 389 employees who received at least one dose of the BNT162b2 vaccine between March 12 and 31, 2021, and underwent IGRA participated in this study. We excluded 68 participants who did not undergo blood tests at any point in time. The characteristics of the 321 included participants are presented in Table 1. The median age of the participants was 38 (range, 23–64) years, and the number of female participants (n = 210, 65.4%) was higher than that of male participants. Most of the participants reported no underlying medical problems. Only 10 (3.1%) participants reported active smoking, and 28 (8.7%) reported past smoking. A total of 179 (55.7%) participants reported social drinking, and 62 (19.3%) reported daily alcohol consumption. Twelve participants who answered that they had been diagnosed with COVID-19 or showed a high titer of anti-nucleocapsid SARS-CoV-2 IgG antibodies (10.0 AU/mL <) during the observation period were analyzed separately; the other 309 participants were analyzed first.

**Table 1:** Characteristics of the study participants who had underwent a series of blood collections.

Characteristics	Total (n = 321)		Males (n = 111)		Females (n = 210)		P value
Age (years, median [range])	38	[23–64]	39	[23–63]	38	[23–64]	0.1124
–29 (%)	54	(16.8)	9	(8.1)	45	(21.4)	
30–39 (%)	113	(35.2)	43	(38.7)	70	(33.3)	
40–49 (%)	103	(32.1)	40	(36.0)	63	(30.0)	
50–59 (%)	42	(13.1)	15	(13.5)	27	(12.9)	
60– (%)	9	(2.8)	4	(3.6)	5	(2.4)	
BMI (kg/m^2^, median [range])	21.2	[14.9–38.7]	22.8	[16.5–38.7]	20.6	[14.9–37.3]	< 0.0001
History of smoking							
Never (%)	283	(88.2)	89	(80.2)	194	(92.4)	0.0024
Former (%)	28	(8.7)	18	(16.2)	10	(4.8)	
Current (%)	10	(3.1)	4	(3.6)	6	(2.9)	
Drinking							
No (%)	80	(24.9)	18	(16.2)	62	(29.5)	0.0013
Social (%)	179	(55.8)	61	(55.0)	118	(56.2)	
Regular (%)	62	(19.3)	32	(28.8)	30	(14.3)	
Occupation type							
Physician (%)	93	(29.0)	66	(59.5)	27	(12.9)	< 0.0001
Nurse (%)	123	(38.3)	5	(4.5)	118	(56.2)	
Other paramedical (%)	77	(24.0)	30	(27.0)	47	(22.4)	
Nonmedical (%)	28	(8.7)	10	(9.0)	18	(8.6)	
Comorbidity							
Allergic disease except for asthma (%)	31	(9.7)	16	(14.4)	15	(7.1)	0.0463
Asthma (%)	25	(7.8)	6	(5.4)	19	(9.0)	0.2815
Hypertension (%)	12	(3.7)	7	(6.3)	5	(2.4)	0.1181
Dyslipidemia (%)	12	(3.7)	6	(5.4)	6	(2.9)	0.3530
Diabetes mellitus (%)	6	(1.9)	1	(0.9)	5	(2.4)	0.6684
COVID-19 (%)	5	(1.6)	1	(0.9)	4	(1.9)	0.6635

Health care workers were classified as either medical or nonmedical staff. The medical staff included nurses, physicians, pharmacists, clinical laboratory technicians, radiologists, and medical students. Nonmedical staff included administrative workers, medical clerks, and other staff members who did not have direct contact with patients.

*BMI* body mass index.

### Cellular immune responses and humoral responses to vaccination

**Figure 1:**
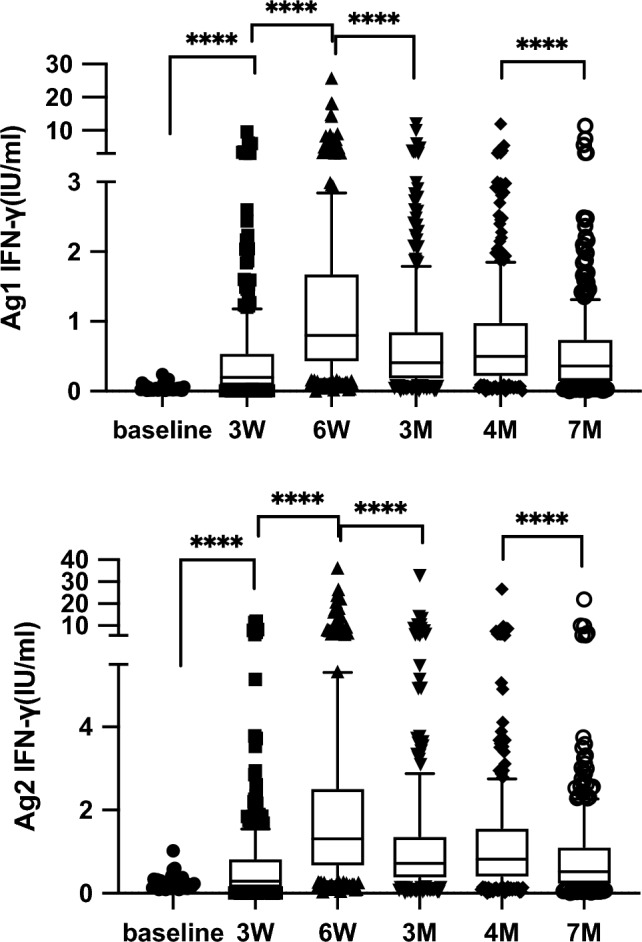
Dynamics of the cellular immune response as the IFN-γ level changes. Blood samples were collected and stimulated with Ag1 (upper) and Ag2 (lower) derived from the SARS-CoV-2 spike protein before and after full vaccination. The time-dependent change of IFN-γ levels, induced by the incubation of lymphocytes with SARS-CoV-2-specific epitopes of CD4 + (Ag1) or CD4 + and CD8 + (Ag2) T cells, were demonstrated. Baseline indicates before the first dose of vaccination, and 3 W means before the second dose. Then IFN-γ levels were also measured in blood samples 3 weeks (6 W), 2 months (3 M), 3 months (4 M), and 6 months (7 M) after the second vaccination. Box plots show the median and interquartile range associated with the min and max. The Friedman test was used to calculate the *P* values (****P < 0.0001).

SARS-CoV-2-specific T-cell responses were measured via IGRA to evaluate cellular immunity. We measured IFN-γ levels using the Ag1 tubes which consist of epitopes of CD4 + T cells derived from the S1 subunit of the spike protein and the Ag2 tubes which consist of epitopes of CD4 + and CD8 + T cells derived from the S1 and S2 subunits. The time course of IFN-γ levels in response to Ag1 and Ag2 is shown in Fig. 1. At baseline, 2 participants and 18 participants were above the cutoff levels for Ag1 and Ag2, respectively. Three weeks after full vaccination (6 W), all participants showed elevated IFN-γ levels from baseline, and 283 (91.6%) and 296 (95.8%) participants showed IFN-γ levels above the cutoff levels for Ag1 and Ag2. The median Ag1 and Ag2 levels were 0.798 AU/mL (interquartile range [IQR], 0.417–1.69) AU/mL and 1.309 (IQR, 0.648–2.53) AU/mL, respectively. Between 2 months (3 M) and 3 months (4 M) after the full vaccination, no significant decrease in IFN-γ levels was observed for either Ag1 or Ag2. At 6 months after full vaccination (7 M), the median Ag1 and Ag2 levels were 0.360 (IQR, 0.130–0.750) AU/mL and 0.520 (IQR, 0.210–1.12) AU/mL, respectively. A total of 227 (73.4%) and 257 (83.1%) participants still showed IFN-γ levels above the cutoffs for Ag1 and Ag2. The decrease in Ag2 was 60.0% from 6 W. Interestingly, there were 43 participants (13.9%) and 42 participants (13.5%) who further showed increased IFN-γ levels in response to Ag1 and Ag2 from 6 W to 7 M despite no evidence of SARS-CoV-2 infection based on the questionnaire or elevation of anti-nucleocapsid antibodies.

**Figure 2:**
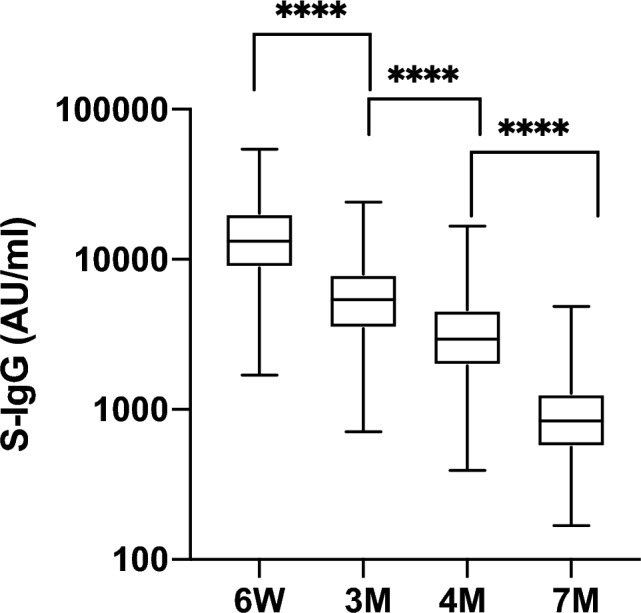
Dynamics of the anti-spike SARS-CoV-2 antibody response after full vaccination with two doses of the BNT162b2 vaccine. Anti-spike SARS-CoV-2 antibody levels were measured in blood samples at 6 W, 3 M, 4 M, and 7 M. Box plots show the median and quartiles. The Friedman test was used to calculate the *P* values (****P < 0.0001).

Regarding humoral immune responses, all participants showed increased levels of anti-spike IgG antibodies above the cutoff level (50.0 AU/mL) at 6 W, with a median level of 13,200 (IQR, 8810–20,200) AU/mL. However, at 7 M, the median level was 839 AU/mL (IQR, 562–1280 AU/mL). Hence, a 93.6% decrease in the antibody titer was observed after 6 months (Fig. 2).

### Correlation between the humoral and cellular immune responses after vaccination

**Figure 3:**
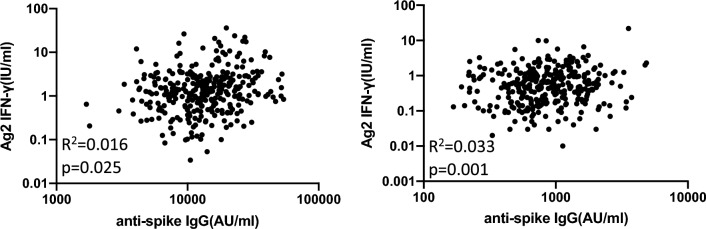
Correlation between humoral and cellular responses 3 weeks (left) and 6 months (right) after the second dose of the BNT162b2 mRNA vaccine. Scatter plot of specific IFN-γ responses in the Ag2 tube and anti-spike IgG antibody levels over time following vaccination. Pearson’s correlation coefficients and P values are indicated.

The time-dependent attenuation of anti-spike IgG antibody levels was faster than that of IFN-γ levels measured via IGRA, as shown in Figs. 1 and 2. We compared the correlation between anti-spike IgG antibody and IFN-γ levels in response to Ag2 at 6 W and 7 M (Fig. 3). Anti-spike IgG antibody levels were attenuated in all participants, whereas some participants (13.5%) showed elevated IFN-γ levels from 6 W to 7 M. These correlations were very weak at 6 W (R^2^ = 0.016, *P* = 0.025). The regression coefficient at 7 M was increased but still weak compared to that at 6 W (R^2^ = 0.033, *P* = 0.001).

### Factors associated with the levels of IFN-γ induced by Ag1 and Ag2 6 months after full vaccination

To clarify the epidemiological and clinical factors that can influence long-lasting cellular immunity induced by vaccination, we performed a multiple regression analysis of IFN-γ levels at 7 M. The factors included blood examination, self-reported adverse reactions, and epidemiological backgrounds. The results of univariate analysis are shown in Supplementary Tables S1 and S2. The results of the multiple regression analysis are shown in Table 2. Age (*P* = 0.047), Ag1 levels at 3 W (*P* < 0.001), and Ag1 levels at 6 W (*P* < 0.001) were significantly associated with Ag1 levels at 7 M, while age (*P* = 0.017), dyslipidemia (*P* = 0.039), focal adverse reactions only (*P* = 0.020), lymphocyte (*P* = 0.012) and monocyte (*P* = 0.010) counts, Ag2 levels at 3 W (*P* < 0.001), and Ag2 levels at 6 W (*P* < 0.001) were significantly associated with Ag2 levels at 7 M. As expected, Ag1 and Ag2 levels were strongly correlated with each other at 7 M. Interestingly, Ag1 and Ag2 levels at 3 W showed a stronger correlation with the level at 7 M than with that at 6 W (partial regression coefficient, 1.366 vs. 1.130 for Ag1 and 1.315 vs. 1.071 for Ag2).

**Table 2:** Multiple regression analysis of factors associated with the levels of IFN-γ induced by Ag1 and Ag2 responses 6 months after administration of the BNT162b2 vaccine.

Explanatory variables	Partial regression coefficient	95% Confidence Interval		*P* value
Ag1				
Age	**1.015**	**1.000**	**1.030**	**0.0473**
Sex (female)	0.788	0.571	1.086	0.1451
Na (mEq/L, pre)	1.042	0.948	1.145	0.3898
Cl (mEq/L, pre)	1.056	0.976	1.142	0.1744
Triglyceride (mg/dL, post 1st dose)	0.999	0.998	1.001	0.3310
Activated partial thromboplastin time (s, post 1st dose)	0.966	0.919	1.015	0.1725
Anti-spike IgG (AU/mL, post 2nd dose)	1.000	1.000	1.000	0.6152
Drinking (daily)	1.292	0.935	1.786	0.1201
Comorbidity (dyslipidemia)	0.736	0.380	1.426	0.3625
Focal adverse reactions only (post 2nd dose)	0.739	0.524	1.041	0.0835
Neutrophils (every 100/μL, post 1st dose)	0.999	0.987	1.011	0.8294
Basophils (every 100/μL, post 1st dose)	0.590	0.338	1.030	0.0633
Ag1 (3 weeks after 1st dose)	**1.366**	**1.213**	**1.537**	** < 0.0001**
Ag1 (3 weeks after 2nd dose)	**1.130**	**1.065**	**1.200**	**0.0001**
Ag2				
Age	**1.016**	**1.003**	**1.030**	**0.0176**
Sex (female)	0.850	0.583	1.239	0.3967
Na (mEq/L, post 1st dose)	0.979	0.893	1.074	0.6582
Cl (mEq/L, post 1st dose)	1.033	0.958	1.115	0.3943
Hemoglobin (g/dL, post 1st dose)	1.084	0.953	1.233	0.2168
Platelet (× 10^4^/μL, post 1st dose)	1.009	0.984	1.035	0.4789
IgG Titer (AU/mL, post 2nd dose)	1.000	1.000	1.000	0.9206
Comorbidity (dyslipidemia)	**0.532**	**0.292**	**0.969**	**0.0393**
Focal adverse reactions only (post 2nd dose)	**0.680**	**0.492**	**0.940**	**0.0198**
Neutrophils (every 100/μL, post 1st dose)	0.999	0.987	1.011	0.8602
Lymphocytes (every 100/μL, post 1st dose)	**1.036**	**1.008**	**1.065**	**0.0115**
Monocytes (every 100/μL, post 1st dose)	**0.821**	**0.706**	**0.954**	**0.0101**
Basophil (every 100/μL, post 1st dose)	0.677	0.390	1.174	0.1643
Ag2 (3 weeks after 1st dose)	**1.315**	**1.190**	**1.454**	** < 0.0001**
Ag2 (3 weeks after 2nd dose)	**1.071**	**1.038**	**1.105**	** < 0.0001**

Factors significantly associated with the levels of IFN-γ (*P* < 0.05) in Ag1 or Ag2 are highlighted in bold.

### Factors associated with a positive IFN-γ response measured via IGRA 6 months after full vaccination

Multivariate logistic regression analysis was performed to assess the factors associated with a positive response (≥ 0.15 IU/mL IFN-γ) in SARS-CoV-2-specific cellular immunity at 7 M (Table 3). Ag1 levels at 3 W and 6 W were significantly associated with a positive response (odds ratio [OR], 3.45; 95% confidence interval [95% CI] 1.45–10.26; *P* = 0.01; OR, 1.71; 95% CI 1.16–2.77; *P* = 0.02, respectively), whereas focal adverse reactions only (OR, 0.34; 95% CI 0.14–0.83; *P* = 0.02), lymphocyte and monocyte counts for every 100/μL (OR, 1.11; 95% CI 1.01–1.22; *P* = 0.04 and OR, 0.55; 95% CI 0.33–0.88; *P* = 0.02, respectively), and Ag2 levels at 3 W (OR, 7.34; 95% CI 2.05–39.01; *P* = 0.008) were significantly associated with Ag2 levels at 7 M. Consistent with the results of multiple regression analysis, previously measured Ag1 and Ag2 levels were associated with each other at 7 M, and Ag1 and Ag2 levels had a stronger association with a positive IFN-γ response at 3 W than at 6 W.

**Table 3:** Logistic regression analysis of factors associated with positive levels of IFN-γ (≧0.15 IU/ml IFN-γ) measured via IGRA 6 months after the administration of the BNT162b2 vaccine.

Explanatory variables	Odds ratio	95% Confidence Interval		*P* value
Ag1				
Age	1.02	0.99	1.05	0.2387
Sex (female)	0.59	0.22	1.56	0.2953
Chloride (mEq/L, prevaccination)	1.17	1.00	1.38	0.0565
Creatinine (mg/dL, post 1st dose)	2.48	0.12	55.36	0.5605
C reactive protein (mg/dL, post 1st dose)	0.10	0.01	0.74	0.0921
Focal adverse reactions only (post 2nd dose)	0.59	0.29	1.24	0.1598
Neutrophils (every 100/μL, post 1st dose)	1.00	0.97	1.03	0.9846
Basophils (every 100/μL, post 1st dose)	0.37	0.10	1.34	0.1305
Ag1 (3 weeks after 1st dose)	**3.45**	**1.45**	**10.26**	**0.013**
Ag1 (3 weeks after 2nd dose)	**1.71**	**1.16**	**2.77**	**0.0175**
Ag2				
Age	1.00	0.96	1.05	0.9577
Sex (Female)	1.08	0.31	3.76	0.9068
Sodium (mEq/L, post 1st dose)	1.15	0.88	1.51	0.3071
Triglycerides (mg/dL, post 1st dose)	1.00	0.99	1.00	0.2581
Hemoglobin (g/dL, post 1st dose)	1.42	0.97	2.11	0.0756
IgG Titer (AU/mL, post 2nd dose)	1.00	1.00	1.00	0.6624
Focal adverse reactions only (post 2nd dose)	**0.34**	**0.14**	**0.83**	**0.0176**
Neutrophils (every 100/μL, post 1st dose)	0.99	1.00	1.00	0.6967
Basophils (every 100/μL, post 1st dose)	0.64	0.98	1.01	0.5221
Lymphocytes (every 100/μL, post 1st dose)	**1.11**	**1.01**	**1.22**	**0.0358**
Monocytes (every 100/μL, post 1st dose)	**0.55**	**0.33**	**0.88**	**0.0158**
Ag2 (3 weeks after 1st dose)	**7.34**	**2.05**	**39.01**	**0.0076**
Ag2 (3 weeks after 2nd dose)	1.27	0.96	1.89	0.1783

Factors significantly associated with the positive response (*P* value < 0.05) in Ag1 or Ag2 are highlighted in bold.

### Dynamics of cellular immune responses in participants who showed positive cellular immune responses without positive anti-nucleocapsid antibodies at baseline

**Figure 4:**
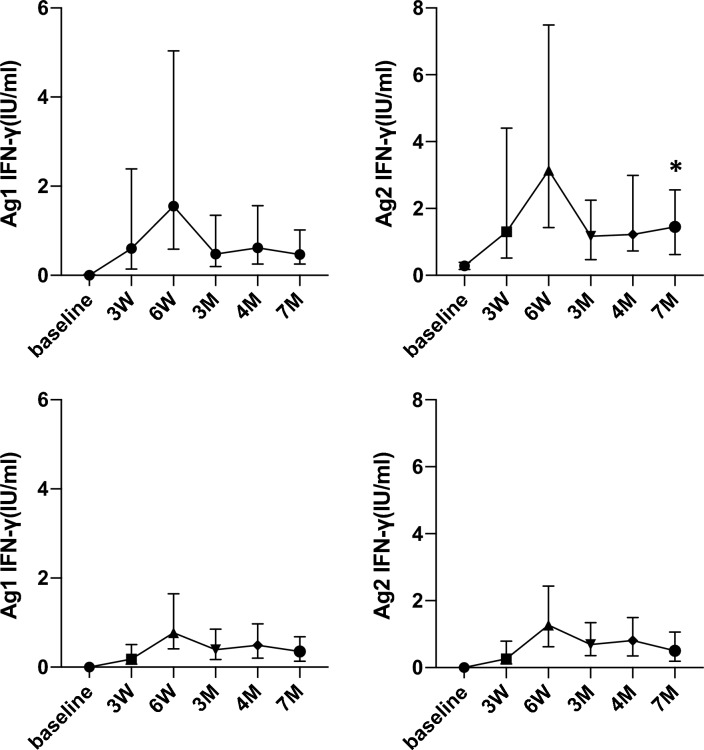
Kinetics of the IFN-γ levels (Ag1; left, Ag2; right) in the participants who showed positive cellular immune responses without positive anti-nucleocapsid antibody levels at baseline (n = 18, upper). Dynamics of the cellular immune response in the participants who showed negative cellular immune responses are also shown (n = 291, lower). At 7 M, IFN-γ levels in response to Ag2 in the participants with positive cellular immune responses without positive anti-nucleocapsid antibody levels at baseline were significantly elevated compared with those in the other participants (*P < 0.05).

At baseline, 2 participants (also showing a positive Ag2 response) and 18 participants were above the cutoff levels for Ag1 and Ag2 without positive anti-nucleocapsid antibodies. Their median age was 33 years (range, 23–57), their body mass index was 20.5 (18–25.8), and they had no episodes of COVID-19 infection. Data on the cellular immune responses are shown in Fig. 4. The median IFN-γ level in response to Ag2 was significantly increased at 7 M compared with that in the other participants (median level, 1.45 vs. 0.50, IQR, 0.62–2.56 vs. 0.19–1.065, *P* < 0.001). Notably, the median value of the IFN-γ levels in response to Ag2 was slightly increased at 7 M compared to 4 M (median level, 1.23 vs. 1.45, IQR, 0.73–2.99 vs. 0.62–2.55, Fig. 4), whereas IFN-γ levels and the levels of anti-spike antibodies in response to Ag1 were decreased at 7 M and showed no significant increase compared with those of the other participants.

### Dynamics of vaccine-associated immune responses in participants with a positive anti-nucleocapsid antibody titer prevaccination

We analyzed immune responses in participants with a positive anti-nucleocapsid antibody titer prevaccination. Five participants were selected, and three had a history of COVID-19 infection with no treatment needed. The time course of their immune responses is described in Table 4. One patient had a positive response for both Ag1 and Ag2 at baseline. All patients showed a positive cellular immune response prior to the second vaccination (3 W). Interestingly, four participants (80%) exhibited a significant decline in the cellular immune response at 6 W compared to 3 W. At 7 M, participants still had positive IFN-γ levels, and both anti-spike IgG antibody and IFN-γ levels were significantly elevated in these participants compared with those with no prior elevation of anti-nucleocapsid antibody levels (*P* < 0.01).

**Table 4:** Characteristics and dynamics of humoral and cellular immune responses in participants with a positive anti-nucleocapsid antibody titer before the first vaccination.

Age (years)	Sex	Previous COVID-19 infection	IFN-γ (Ag1) IU/mL						IFN-γ (Ag2) IU/mL						Anti-spike IgG (AU/mL)			
			baseline	3 W	6 W	3 M	4 M	7 M	baseline	3 W	6 W	3 M	4 M	7 M	6 W	3 M	4 M	7 M
35	F	−	0.021	11.2	5.1	5.89	2.95	7.25	0.05	4.5	3.7	5.92	3.73	6.76	14,500	10,100	6540	1990
47	F	+	0.965	2.26	1.1	2.84	1.37	1.19	1.28	3.12	1.64	3.7	2.01	1.59	22,900	11,000	8560	4160
45	F	+	0.031	0.852	1.66	0.4	2.4	1.19	0.028	0.842	1.72	1.37	2.23	1.66	19,100	10,200	6170	1860
23	F	−	0.001	0.433	0.3	0.32	0.33	0.21	N.D	0.703	0.448	0.36	0.72	0.26	37,600	16,700	10,800	2020
34	M	+	0.101	1.95	1.78	1.46	1.84	0.89	0.299	3.85	3.48	2.81	3.07	1.84	11,600	4030	2520	1280

COVID-19 infection was based on the answers provided in the questionnaire.

*N.D.* not detected.

## Discussion

We monitored and analyzed the cellular immune response elicited by the BNT162b2 mRNA vaccine until 6 months after the second vaccination. IFN-γ, induced by the incubation of lymphocytes with SARS-CoV-2-specific epitopes of CD4 + (Ag1) or CD4 + and CD8 + (Ag2) T cells, peaked at 6 W and typically decreased between 6 W, 3 M, and 7 M. The degree of reduction in IFN-γ levels in response to Ag1 and Ag2 from 6 W to 7 M was 54.9% and 60.3%, respectively, whereas that of the anti-spike IgG antibody levels was 93.7%. This observation that the IFN-γ titer tends to persist at 6 months after full vaccination compared with the anti-spike antibody titer is consistent with another similar study using the BNT162b2 mRNA vaccine^23,24^.

The attenuation of cellular and humoral immune responses was similar to that reported in previous studies conducted with health care workers^22^. A similar decline in anti-spike IgG antibody levels was also observed in other studies with the BNT162b2 mRNA vaccine^19,25^, except among elderly people and people with immunosuppressive conditions. Between 3 and 4 M, the anti-spike antibody titer decreased by 44%, and the IFN-γ titer in response to Ag2 decreased by 5.2%, while between 4 and 7 M, the antibody titer decreased by 71.9%, and the IFN-γ titer decreased by 27.6%. Our data demonstrated that the decline in both humoral and cellular immune responses was relatively high between 4 and 7 M compared with that between 3 and 4 M. This observation is consistent with evidence that the rate ratio for SARS-CoV-2 infection and severe diseases increased 6 months after full vaccination compared with that after 4 months^26^. These results emphasize the need to administer a booster dose to induce sufficient immunity for disease prevention^27,28^.

Regarding the correlation of the levels of anti-spike IgG antibodies and IFN-γ, we observed only a weak correlation at both 3 M and 7 M. Another study conducted with health care workers also showed a stronger correlation between these levels 6 months after full vaccination compared with that 3 months after vaccination^21^, but correlations at both times seemed stronger than those in our study. Another study in Japan also noted a very weak correlation between these levels, similar to that in our study^29^; this difference may be attributed to differences in the population. Between 4 and 7 M, six participants were positive for anti-nucleocapsid antibodies (excluded from the main analysis), with suspicion of breakthrough infection. Their humoral and cellular immune responses were not impaired at 4 M compared with those of the other participants, and participants with confirmed COVID-19 infection between 4 and 7 M had mild symptoms and did not require antiviral or steroid therapy (data not shown).

At baseline, IFN-γ levels in response to Ag2 were above the cutoff in 18 (5.8%) participants, without an increase in anti-nucleocapsid antibodies. They still had significantly higher IFN-γ levels in response to Ag2 but the levels response to Ag1 were not significant at 7 M compared with those below the cutoff level. In fact, 6/18 (33.3%) participants had higher IFN-γ levels at 7 M than at 4 M, whereas we found the same tendency in 79/291 (27.1%) participants with levels below the cutoff at baseline, suggesting that circulating activated memory T cells specific for SARS-CoV-2 could be maintained longer in participants with positive responses to Ag2 at baseline. Our results suggest that blood CD8 + T cells in some people already showed reactivity with epitopes from the SARS-CoV-2 spike protein before vaccination, and they had a long-lasting cellular immune response. This early reactivity could presumably be attributed to past exposure to seasonal coronaviruses, which have conserved peptides^30,31^. Other studies have demonstrated that these cells might be important for immune defense against SARS-CoV-2, as blood CD8 + T cells with these conserved epitopes are associated with mild symptoms in patients with COVID-19 infection^30^ and long-lasting protective immunity^32^. Considering that cellular immune responses, particularly CD8 + T-cell responses, contribute to protection against severe SARS-CoV-2 infection^16^, people with natural CD8 + T-cell responses to SARS-CoV-2 may be protected against developing severe disease. Further studies are needed to clarify the distinct phenotype of T cells that contributes to evoking effective cellular immunity.

Participants with a positive anti-nucleocapsid antibody titer at baseline showed a positive cellular immune response with the first vaccination, but 80% of them showed a decline in the response to the second vaccine. Similar results regarding vaccination with subjects who recovered from COVID-19 infection have been reported both in cellular and humoral immune responses^33,34^. Regarding humoral responses, memory B-cell responses one week after the first vaccination were boosted in people who had recovered from COVID-19 infection, while naive individuals required two doses to reach comparable memory B-cell levels^34^. Regarding cellular responses, naive individuals exhibited higher SARS-CoV-2-specific CD4 + T-cell activation and proliferation than individuals who had recovered from COVID-19 infection 2 weeks after the second vaccination^33^. Consistent with previous studies, our study implicates that the first vaccination works as a booster and that no further immune response is observed after the second vaccination in participants with prior COVID-19 infection, and the response decreases during the time course. Regulatory T cells may be also involved in this effect^33^. Although it is questionable whether memory T cells are activated sufficiently with one vaccination in individuals with prior COVID-19 infection compared with those in fully vaccinated naive individuals, in our results, both cellular and humoral responses were relatively high in individuals with prior COVID-19 infection compared with those in naive individuals at 7 M.

To identify the factors that could predict a strong cellular immune response, we performed a multivariate analysis. Older age and dyslipidemia were significantly associated with a strong cellular immune response but not with positive responses at 7 M. Focal adverse reactions after the second vaccination and the blood lymphocyte and monocyte counts were associated with strong and positive cellular immune responses at 7 M. The younger age is associated with the stronger humoral response at earlier stages after full vaccination. However, it is controversial whether the difference is maintained after 6–9 months^35,36^. Other authors demonstrated that low high-density lipoprotein cholesterol or high triglycerides were not associated with the results of IGRA for tuberculosis^37^. Regarding adverse effects, our previous study demonstrated that participants reporting stronger reactions after the second dose than after the first dose showed significantly higher levels of the anti-spike IgG antibody^15^, implying that relatively strong adverse reactions are possibly associated with both strong humoral and cellular immunity induced by BNT162b2 vaccines. In another study, vaccinated individuals with general fatigue and fever as adverse events had stronger cellular immune responses than those without them 8 weeks after the second vaccination^29^. Our study suggests that systemic adverse reactions after the second dose and the lymphocyte count at baseline were positively associated with strong and positive cellular immune responses at 7 M. Other studies demonstrated that humoral responses correlated with higher B-, NK- and CD4- cell counts in the vaccination period, and a low CD4 + /CD8 + ratio was correlated with cellular response failure in patients after allogenic hematopoietic stem cell transplantations^38,39^. Further studies are needed to determine whether the number of lymphocytes correlates with that of memory CD4 + and CD8 + T cells that can react with SARS-CoV-2 epitopes. As expected, Ag1 and Ag2 levels were strongly associated with each other at 7 M, but Ag1 and Ag2 levels at 3 W had a stronger association with those at 7 M than at 6 W. These results suggest that the first dose is a better predictor of long-lasting cellular immunity and memory T-cell activity than the second dose, which could induce strong adaptive immunity but result in temporally strong immune responses, and differences between individuals might be more remarkable after the second dose than those after the first dose. We have to note that other factors we didn’t investigate might affect our results. For example, in IGRA, HLA is thought to have an essential role through antigen recognition and interaction between antigen-presenting cells and T cells, which might influence our results. In tuberculosis, HLA-DR polymorphism was associated with IGRA sensitivity^40^. Another study demonstrated that HLA-DR was a marker of recently divided CD4 T cells upon *M. tuberculosis* antigen exposure^41^. For SARS-CoV-2 vaccination, cellular response failure correlated with higher HLA-DR + T cell levels in recipients of allogeneic hematopoietic cell transplantation^38^.

This study had several limitations. First, this was a single-center study using the BNT162b2 vaccine and involving health care workers. It remains uncertain whether our findings can be generalized to other populations and COVID-19 mRNA vaccines. Second, we measured levels of anti-spike antibodies instead of SARS-CoV-2 neutralizing antibodies. Neutralizing antibodies are associated with protection^42^. Anti-spike antibodies are known to be correlated with SARS-CoV-2 neutralizing antibodies postinfection^43^ and postvaccination^22^, and we confirmed their correlation in small samples after full vaccination (Supplementary Fig. S1). Third, we selected participants with positive anti-nucleocapsid antibodies or with symptoms of SARS-CoV-2 infection based on the questionnaire and possible asymptomatic infections who had negative levels of anti-nucleocapsid antibodies during the examination period. Between 4 and 7 M, Japan was in the fifth wave of the SARS-CoV-2 epidemic due to the Delta (B.1.617.2) variant, and asymptomatic infection might have been more frequent in this period than in other periods.

In conclusion, we observed a substantial decline in both cellular and humoral immune responses 6 months after the administration of BNT162b2 vaccines, although the dynamics of cellular immune responses were somewhat distinct from those of humoral immune responses. Our study emphasizes the need for booster vaccination, and future studies are needed to clarify the dynamics of cellular immune responses after booster vaccination and the clinical significance of the coordination between humoral and cellular immunity.

## Methods

### Study design and participants

This prospective observational study was conducted at the University of Tokyo Hospital, Tokyo, Japan. In Japan, SARS-CoV-2 vaccination with the BNT162b2 mRNA vaccine for health care workers was made available in February 2021. Health care workers who were requested to receive the first dose of the BNT162b2 mRNA vaccine at the University of Tokyo Hospital from March 12 to March 31, 2021, were invited to participate in this study. Participants were included if they indicated their willingness to participate prior to the first vaccination. There were no exclusion criteria regarding the health conditions of the participants. This study was conducted in accordance with the Declaration of Helsinki and approved by the ethics committee of the University of Tokyo Hospital (approval number: 2020353NI). Written informed consent was obtained from each participant at the start of the study.

### Data collection and SARS-CoV-2 antibody immunoassays

Blood samples and clinical information of the study participants based on the questionnaire were collected prior to the first (baseline) and second (3 W) doses, 3 weeks after the second dose (6 W), 2 months after the second dose (3 M), 3 months after the second dose (4 M), and 6 months after the second dose (7 M), as previously described in detail^15^. Briefly, the following characteristics were collected via an online questionnaire at the first sample collection: participant age, race, sex, height, weight, occupation type, comorbidities, smoking and drinking status, and exposure to outpatients or inpatients. Self-reported information on the history of COVID-19 infection or close contact with confirmed COVID-19 patients was collected at each visit for blood sample collection. In addition, information on adverse reactions after the first and second doses of vaccination was collected. We measured anti-spike IgG antibody levels (SARS-CoV-2 IgG II Quant assay, Abbott Architect, U.S.) using a CLIA, and the positive cutoff antibody level was defined as 50.0 AU/mL according to the manufacturer’s instructions. We also measured anti-nucleocapsid SARS-CoV-2 IgG antibody levels using an automated CLIA (iFlash3000, YHLO, Shenzhen, China) to assess COVID-19 infection status. A positive cutoff index was defined as 10.0 AU/mL, according to the manufacturer’s instructions. In addition, we measured the counts and levels of the following before the first and second doses: complete blood cell counts, renal function, liver function, electrolytes, lipids, C-reactive protein, D-dimer, prothrombin time, and activated partial thromboplastin time. Data on epidemiological characteristics and adverse reactions to vaccination were collected using an online questionnaire.

### Measurement of the SARS-CoV-2-specific T-cell response

The T-cell responses in the peripheral blood were evaluated via IGRA. We used QuantiFERON SARS-CoV-2 Research Use Only, including blood collection tubes from the SARS-CoV-2 starter and control sets (Qiagen, Hilden, Germany). The kit consisted of Ag1, Ag2, negative control, and positive control tubes. The Ag1 tubes consisted of epitopes of CD4 + T cells derived from the S1 subunit of the spike protein. The Ag2 tubes consisted of epitopes of CD4 + and CD8 + T cells derived from the S1 and S2 subunits. Whole blood was incubated with SARS-CoV-2 epitopes in tubes at 37 °C for 16–24 h, and the IFN-γ concentration in plasma was measured by enzyme-linked immunosorbent assay according to the manufacturer’s instructions. The cutoff for this assay was determined using data from 20 subjects who had tested as nonreactive for SARS-CoV-2 with an authorized RT‒PCR test or FDA-authorized serology test and 20 donors who were fully vaccinated (between 2 and 16 weeks after full vaccination) with an FDA EUA-authorized vaccine^44^. With a cutoff value of 0.15 IU/mL, the test sensitivity and specificity were determined to be 98.3% and 100%, respectively, according to the manufacturer’s instructions. The individual IFN-γ concentrations were calculated by subtracting from the IFN-γ titer in the negative control.

### Multiple regression analysis and sensitivity analysis to assess the factors associated with log-transformed IFN-γ levels

We selected the explanatory variables as follows: First, we selected variables with biological plausibility, such as age, sex, smoking history, and drinking history. Second, we performed a univariate analysis with the other variables (i.e., adverse reactions after both vaccinations, comorbidities, and blood test results) and identified the factors with a P value below 0.1 for regression coefficients. If the items from the first and second blood draws were the same, those from the second blood draw were retained. Third, we entered these variables in the final model.

### Statistical analysis

Information from the participants who had undergone all six blood tests was used for the analysis. The Levene test was used to check for equality of variances. The Brunner–Munzel and Kruskal–Wallis tests were used to compare continuous variables. Fisher’s exact and chi-square tests were used for other categorical variables. We performed a multiple regression analysis to assess the factors associated with IFN-γ levels in response to Ag1 and Ag2. We explored the factors associated with a positive SARS-CoV-2-specific cellular immunity response using multiple logistic regression analysis in a similar manner. We performed all statistical analyses using R 4.0.3^45^ with the “lawstat”^46^ and “tidyverse”^47^ packages, and created figures by using Prism 8 (GraphPad). All tests were two-tailed, and a P value < 0.05 was considered statistically significant.

### Ethical approval

This study was conducted in accordance with the Declaration of Helsinki and approved by the ethics committee of the University of Tokyo Hospital (approval number: 2020353NI). Written informed consent was obtained from each participant at the start of the study.

## Supplementary Information

Supplementary Information.

## Data Availability

All data are available within the manuscript, figures, and tables, including the supplemental material.
