# Supplementary material for: Waning cellular immune responses and predictive factors in maintaining cellular immunity against SARS-CoV-2 six months after BNT162b2 mRNA vaccination
Source: Sci Rep. 2023 Jun 13;13:9607. doi: 10.1038/s41598-023-36397-6 (PMC10263378; doi:10.1038/s41598-023-36397-6)
Supplement: Supplementary file 1 — Supplementary Information. [file 41598_2023_36397_MOESM1_ESM.docx]

**Supplementary Information**

**Title**

**Waning cellular immune responses and predictive factors in maintaining cellular immunity against SARS-CoV-2 six months after BNT162b2 mRNA vaccination**

Authors: Takashi Ishii MD, PhD^1^, Kensuke Hamada MD, PhD^1^, Daisuke Jubishi MD, PhD^2^, Hideki Hashimoto MD, PhD^2^, Koh Okamoto MD, MS, PhD ^2^, Naoko Hisasue MT, MS^1^, Mitsuhiro Sunohara MD, PhD^1^, Minako Saito MD, PhD^1^, Takayuki Shinohara MD^2^, Marie Yamashita MD^2^, Yuji Wakimoto MD^2^, Amato Otani MD^2^, Mahoko Ikeda MD, PhD^2, 3^, Sohei Harada MD, PhD^3^, Shu Okugawa MD, PhD^2^, Kyoji Moriya MD, PhD^1^, Shintaro Yanagimoto MD, PhD^1^.

Affiliation:

^1^ Division for Health Service Promotion, The University of Tokyo, Tokyo, Japan.

^2^ Department of Infectious Diseases, The University of Tokyo Hospital, Tokyo, Japan.

^3^ Department of Infection Control and Prevention, The University of Tokyo Hospital, Tokyo, Japan.

**Supplementary Table 1.**

**Univariate analysis of factors associated with the levels of IFN-γ elicited by Ag.1 6 months after the BNT162b2 vaccine administration.**

| Variable | Regression coefficient | 95% confidence interval | | P value |
| --- | --- | --- | --- | --- |
| Age | 1.011 | 0.996 | 1.026 | 0.160 |
| BMI (kg/m^2^) | 1.028 | 0.989 | 1.069 | 0.165 |
| Smoking currently | 0.861 | 0.345 | 2.149 | 0.748 |
| Never have smoked | 1.085 | 0.693 | 1.700 | 0.720 |
| **Drinking daily** | 1.374 | 0.961 | 1.964 | 0.082 |
| Never have drinked | 0.982 | 0.709 | 1.359 | 0.911 |
| Comorbidity (Allergic diseases except for asthma) | 0.803 | 0.499 | 1.291 | 0.363 |
| Comorbidity (Asthma) | 1.403 | 0.826 | 2.382 | 0.209 |
| Comorbidity (Hypertension) | 1.808 | 0.870 | 3.758 | 0.112 |
| **Comorbidity (Dyslipidemia)** | 0.496 | 0.246 | 0.999 | 0.050 |
| Comorbidity (Diabetes mellitus) | 1.252 | 0.467 | 3.355 | 0.654 |
| Focal adverse reactions only (post 1st dose) | 1.029 | 0.773 | 1.370 | 0.844 |
| Systemic adverse reactions (post 1st dose) | 1.194 | 0.893 | 1.596 | 0.230 |
| **Focal adverse reactions only (post 2nd dose)*** | **0.571** | **0.397** | **0.822** | **0.003** |
| **Systemic adverse reactions (post 2nd dose)*** | **1.642** | **1.170** | **2.304** | **0.004** |
| Aspartate aminotransferase (IU/L, pre) | 1.003 | 0.990 | 1.015 | 0.686 |
| Alanine transaminase (IU/L, pre) | 0.999 | 0.991 | 1.007 | 0.854 |
| Creatinine (mg/dL, pre) | 1.658 | 0.657 | 4.188 | 0.283 |
| Blood urea nitrogen (mg/dL, pre) | 1.005 | 0.967 | 1.045 | 0.795 |
| **Na (mEq/L, pre)*** | **1.095** | **1.008** | **1.190** | **0.032** |
| K (mEq/L, pre) | 0.971 | 0.681 | 1.383 | 0.869 |
| **Cl (mEq/L, pre)*** | **1.120** | **1.040** | **1.207** | **0.003** |
| LDL (mg/dL, pre) | 1.001 | 0.997 | 1.006 | 0.530 |
| HDL (mg/dL, pre) | 1.004 | 0.996 | 1.013 | 0.347 |
| **Triglyceride (mg/dL, pre)** | 0.999 | 0.997 | 1.000 | 0.079 |
| Albumin (g/dL, pre) | 0.850 | 0.502 | 1.439 | 0.545 |
| C reactive protein (mg/dL, pre) | 0.778 | 0.348 | 1.738 | 0.539 |
| **Neutrophil (/μL, pre)*** | **0.977** | **0.961** | **0.992** | **0.004** |
| **Lymphocyte (/μL, pre)*** | **1.029** | **1.011** | **1.047** | **0.002** |
| Monocyte (/μL, pre) | 0.984 | 0.890 | 1.088 | 0.748 |
| Eosinophil (/μL, pre) | 1.030 | 0.962 | 1.104 | 0.395 |
| **Basophil (/μL, pre)*** | 0.694 | 0.495 | 0.972 | 0.034 |
| Hemoglobin (g/dL, pre) | 1.028 | 0.931 | 1.135 | 0.589 |
| Platelet (×10^4^/μL, pre) | 0.982 | 0.958 | 1.006 | 0.136 |
| Glucose (mg/dL, pre) | 0.998 | 0.991 | 1.005 | 0.617 |
| Hemoglobin A1C (%, pre) | 0.879 | 0.640 | 1.209 | 0.428 |
| Activated partial thromboplastin time (sec, pre) | 0.962 | 0.913 | 1.014 | 0.150 |
| Prothrombin time (international normalized ratio, pre) | 0.357 | 0.033 | 3.828 | 0.393 |
| D-dimer (μg/mL, pre) | 0.678 | 0.285 | 1.615 | 0.379 |
| Aspartate aminotransferase (IU/L, post 1st dose) | 1.003 | 0.982 | 1.024 | 0.770 |
| Alanine transaminase (IU/L, post 1st dose) | 1.000 | 0.991 | 1.009 | 0.987 |
| Creatinine (mg/dL, post 1st dose) | 1.758 | 0.680 | 4.544 | 0.243 |
| Blood urea nitrogen (mg/dL, post 1st dose) | 1.024 | 0.983 | 1.066 | 0.248 |
| Na (mEq/L, post 1st dose) | 1.064 | 0.978 | 1.159 | 0.150 |
| K (mEq/L, post 1st dose) | 0.937 | 0.618 | 1.421 | 0.760 |
| Cl (mEq/L, post 1st dose) | 1.056 | 0.975 | 1.144 | 0.181 |
| LDL (mg/dL, post 1st dose) | 1.002 | 0.998 | 1.007 | 0.290 |
| HDL (mg/dL, post 1st dose) | 1.006 | 0.997 | 1.014 | 0.188 |
| **Triglyceride (mg/dL, post 1st dose)** | 0.999 | 0.997 | 1.000 | 0.088 |
| Albumin (g/dL, post 1st dose) | 1.090 | 0.654 | 1.817 | 0.739 |
| C reactive protein (mg/dL, post 1st dose) | 0.779 | 0.529 | 1.147 | 0.205 |
| **Neutrophil (/μL, post 1st dose)*** | **0.979** | **0.963** | **0.994** | **0.008** |
| **Lymphocyte (/μL, post 1st dose)*** | **1.029** | **1.011** | **1.047** | **0.002** |
| Monocyte (/μL, post 1st dose) | 0.959 | 0.865 | 1.063 | 0.421 |
| Eosinophil (/μL, post 1st dose) | 1.019 | 0.944 | 1.100 | 0.626 |
| Basophil (/μL, post 1st dose) | 0.770 | 0.546 | 1.086 | 0.135 |
| Hemoglobin (g/dL, post 1st dose) | 1.064 | 0.963 | 1.177 | 0.222 |
| Platelet (×10^4^/μL, post 1st dose) | 0.985 | 0.960 | 1.011 | 0.253 |
| Glucose (mg/dL, post 1st dose) | 0.996 | 0.990 | 1.002 | 0.194 |
| Hemoglobin A1C (%, post 1st dose) | 0.915 | 0.666 | 1.255 | 0.580 |
| **Activated partial thromboplastin time (sec, post 1st dose)** | 0.949 | 0.898 | 1.003 | 0.064 |
| Prothrombin time (international normalized ratio, post 1st dose) | 0.241 | 0.024 | 2.434 | 0.227 |
| D-dimer (μg/mL, post 1st dose) | 0.731 | 0.399 | 1.340 | 0.310 |
| **anti- spike IgG (AU/mL, post 2nd dose)*** | **1.000** | **1.000** | **1.000** | **0.026** |

Factors entered in the final model to perform multiple regression analysis are highlighted in bold. (**P* < 0.05)

**Supplementary Table 2.**

**Univariate analysis of factors associated with the levels of IFN-γ elicited by Ag.2 6 months after the BNT162b2 vaccine administration.**

| Variable | Regression coefficient | 95% confidence interval | | P value |
| --- | --- | --- | --- | --- |
| **Age** | 1.013 | 0.998 | 1.027 | 0.085 |
| BMI (kg/m^2^) | 1.020 | 0.983 | 1.059 | 0.298 |
| Smoking currently | 1.028 | 0.426 | 2.484 | 0.950 |
| Never have smoked | 1.196 | 0.776 | 1.842 | 0.417 |
| Drinking daily | 1.217 | 0.861 | 1.719 | 0.265 |
| Never have drinked | 0.949 | 0.694 | 1.299 | 0.745 |
| Comorbidity (Allergic diseases except for asthma) | 0.739 | 0.468 | 1.168 | 0.195 |
| Comorbidity (Asthma) | 1.472 | 0.884 | 2.450 | 0.137 |
| Comorbidity (Hypertension) | 1.231 | 0.607 | 2.500 | 0.563 |
| **Comorbidity (Dyslipidemia)*** | **0.457** | **0.233** | **0.896** | **0.023** |
| Comorbidity (Diabetes mellitus) | 0.995 | 0.384 | 2.574 | 0.991 |
| Focal adverse reactions only (post 1st dose) | 1.090 | 0.828 | 1.436 | 0.537 |
| Systemic adverse reactions (post 1st dose) | 1.050 | 0.793 | 1.391 | 0.730 |
| **Focal adverse reactions only (post 2nd dose)*** | **0.568** | **0.400** | **0.806** | **0.002** |
| **Systemic adverse reactions (post 2nd dose)*** | **1.709** | **1.234** | **2.366** | **0.001** |
| Aspartate aminotransferase (IU/L, pre) | 1.003 | 0.992 | 1.015 | 0.592 |
| Alanine transaminase (IU/L, pre) | 0.999 | 0.991 | 1.007 | 0.808 |
| Creatinine (mg/dL, pre) | 1.585 | 0.649 | 3.873 | 0.311 |
| Blood urea nitrogen (mg/dL, pre) | 1.010 | 0.973 | 1.049 | 0.599 |
| **Na (mEq/L, pre)*** | **1.115** | **1.029** | **1.208** | **0.008** |
| K (mEq/L, pre) | 1.016 | 0.722 | 1.429 | 0.927 |
| **Cl (mEq/L, pre)*** | **1.115** | **1.038** | **1.198** | **0.003** |
| LDL (mg/dL, pre) | 1.001 | 0.997 | 1.006 | 0.534 |
| HDL (mg/dL, pre) | 1.002 | 0.994 | 1.010 | 0.670 |
| Triglyceride (mg/dL, pre) | 0.999 | 0.998 | 1.001 | 0.434 |
| Albumin (g/dL, pre) | 0.796 | 0.480 | 1.322 | 0.377 |
| C reactive protein (mg/dL, pre) | 0.723 | 0.333 | 1.570 | 0.411 |
| **Neutrophil (/μL, pre)*** | **0.978** | **0.963** | **0.993** | **0.005** |
| **Lymphocyte (/μL, pre)*** | **1.029** | **1.012** | **1.047** | **0.001** |
| Monocyte (/μL, pre) | 0.952 | 0.864 | 1.049 | 0.317 |
| Eosinophil (/μL, pre) | 1.012 | 0.947 | 1.082 | 0.721 |
| **Basophil (/μL, pre)*** | **0.690** | **0.498** | **0.955** | **0.025** |
| Hemoglobin (g/dL, pre) | 1.044 | 0.949 | 1.149 | 0.378 |
| **Platelet (×10^4^/μL, pre)*** | **0.976** | **0.954** | **0.999** | **0.042** |
| Glucose (mg/dL, pre) | 0.998 | 0.991 | 1.004 | 0.502 |
| Hemoglobin A1C (%, pre) | 0.864 | 0.636 | 1.175 | 0.351 |
| Activated partial thromboplastin time (sec, pre) | 0.977 | 0.929 | 1.028 | 0.372 |
| Prothrombin time (international normalized ratio, pre) | 0.289 | 0.029 | 2.844 | 0.286 |
| D-dimer (μg/mL, pre) | 0.732 | 0.317 | 1.691 | 0.464 |
| Aspartate aminotransferase (IU/L, post 1st dose) | 1.007 | 0.987 | 1.027 | 0.512 |
| Alanine transaminase (IU/L, post 1st dose) | 1.001 | 0.992 | 1.010 | 0.900 |
| Creatinine (mg/dL, post 1st dose) | 1.827 | 0.732 | 4.561 | 0.196 |
| Blood urea nitrogen (mg/dL, post 1st dose) | 1.024 | 0.984 | 1.064 | 0.240 |
| Na (mEq/L, post 1st dose) | 1.078 | 0.993 | 1.170 | 0.072 |
| K (mEq/L, post 1st dose) | 0.972 | 0.651 | 1.451 | 0.888 |
| **Cl (mEq/L, post 1st dose)** | 1.068 | 0.989 | 1.153 | 0.093 |
| LDL (mg/dL, post 1st dose) | 1.003 | 0.998 | 1.007 | 0.268 |
| HDL (mg/dL, post 1st dose) | 1.004 | 0.996 | 1.012 | 0.385 |
| Triglyceride (mg/dL, post 1st dose) | 0.999 | 0.998 | 1.001 | 0.430 |
| Albumin (g/dL, post 1st dose) | 1.104 | 0.675 | 1.806 | 0.692 |
| C reactive protein (mg/dL, post 1st dose) | 0.753 | 0.519 | 1.094 | 0.136 |
| **Neutrophil (/μL, post 1st dose)*** | **0.978** | **0.963** | **0.993** | **0.005** |
| **Lymphocyte (/μL, post 1st dose)*** | **1.032** | **1.014** | **1.049** | **0.000** |
| **Monocyte (/μL, post 1st dose)** | 0.913 | 0.828 | 1.008 | 0.071 |
| Eosinophil (/μL, post 1st dose) | 1.005 | 0.933 | 1.081 | 0.905 |
| Basophil (/μL, post 1st dose) | 0.779 | 0.560 | 1.085 | 0.140 |
| **Hemoglobin (g/dL, post 1st dose)** | 1.087 | 0.987 | 1.198 | 0.063 |
| **Platelet (×10^4^/μL, post 1st dose)** | 0.978 | 0.954 | 1.002 | 0.070 |
| Glucose (mg/dL, post 1st dose) | 0.996 | 0.990 | 1.001 | 0.146 |
| Hemoglobin A1C (%, post 1st dose) | 0.886 | 0.653 | 1.202 | 0.435 |
| Activated partial thromboplastin time (sec, post 1st dose) | 0.963 | 0.913 | 1.016 | 0.172 |
| Prothrombin time (international normalized ratio, post 1st dose) | 0.184 | 0.020 | 1.701 | 0.135 |
| D-dimer (μg/mL, post 1st dose) | 0.912 | 0.508 | 1.637 | 0.757 |
| **anti-spike IgG (AU/mL, post 2nd dose)*** | **1.000** | **1.000** | **1.000** | **0.027** |

Factors entered in the final model to perform multiple regression analysis are highlighted in bold. (**P* < 0.05)

Supplementary Figure. Correlation between the SARS-CoV-2 anti-spike antibodies and SARS-CoV-2 neutralizing antibodies (YHLO, Shenzhen, China) after three weeks of the second dose of BNT162b2 mRNA vaccine (n=10). Scatter plot of the titer of anti-spike IgG and neutralizing antibodies. Pearson’s correlation coefficients and P values are indicated.
